# Midfrontal theta power encodes the value of haptic delay

**DOI:** 10.1038/s41598-022-12911-0

**Published:** 2022-05-25

**Authors:** Haneen Alsuradi, Wanjoo Park, Mohamad Eid

**Affiliations:** 1 Tandon School of Engineering, New York University, New York, NY 11201 USA; 2 Engineering Division, New York University Abu Dhabi, Saadiyat Island, Abu Dhabi, 129188 United Arab Emirates

**Keywords:** Engineering, Neuroscience, Somatosensory system

## Abstract

The use of haptic technologies in modern life scenarios is becoming the new normal particularly in rehabilitation, medical training, and entertainment applications. An evident challenge in haptic telepresence systems is the delay in haptic information. How humans perceive delayed visual and audio information has been extensively studied, however, the same for haptically delayed environments remains largely unknown. Here, we develop a visuo-haptic experimental setting that simulates pick and place task and involves continuous haptic feedback stimulation with four possible haptic delay levels. The setting is built using a haptic device and a computer screen. We use electroencephalography (EEG) to study the neural correlates that could be used to identify the amount of the experienced haptic delay. EEG data were collected from 34 participants. Results revealed that midfrontal theta oscillation plays a pivotal role in quantifying the amount of haptic delay while parietal alpha showed a significant modulation that encodes the presence of haptic delay. Based on the available literature, these results suggest that the amount of haptic delay is proportional to the neural activation that is associated with conflict detection and resolution as well as for multi-sensory divided attention.

## Introduction

The introduction of haptic feedback in human–machine interaction systems, such as teleoperation or virtual reality, leads to the potential for timing issues caused by transmission over a computer network or limited computational resources^1^. Haptic delay is the temporal mismatch between the actual haptic feedback event and the expected one. The impact of haptic delay is well studied; it is known that haptic delay leads to deteriorated performance and the potential to compromise the stability of the haptic interface^2^. For instance, delayed haptic feedback can seriously disrupt the task completion time in manipulation^3^, quality of teleoperation^4^, and the perception of physical properties such as stiffness and friction^5^.

The ability of humans to recognize haptic delays may vary substantially based on the application and the type of haptic interaction (vibrotactile versus force feedback, discrete versus continuous force feedback, and passive versus active interaction)^6^. A previous study showed that discrete haptic feedback is noticed as delayed if the delay exceeded 110 ms^6^. In a collaborative virtual environment where haptic data is transmitted bi-directionally, haptic feedback delay could be perceived starting from around 50 ms^7^. It has also been reported that humans do not perceive delays below 30 ms during continuous haptic interaction^8^. In a recent study, the effect of haptic delay for tasks relying on continuous haptic feedback is examined through a human–robot interaction paradigm^9^. Results demonstrated that subjects could recognize haptic delays starting from 20 ms, and at 60 ms and beyond, they clearly perceive the effect of the delay. Understanding the human experience of haptic delay is essential for designing haptic technologies, particularly those intended for use over a computer network.

The integration of neuroscience and haptics inspired an emerging field, named neurohaptics, that strives to understand the complex neural representations provoked in response to touch stimulation^10^. Brain imaging techniques such as fMRI and EEG have the potential to measure brain activities associated with haptic delay to provide neural, real-time assessment of the haptic delay^10,11^. EEG imaging seems superior due to its compatibility with electronic devices, relatively low cost, and high temporal resolution^10^. Previous studies on delay perception with EEG focused primarily on visual delay^12,13^. In addition, previous literature has unraveled the functional roles that the central and midfrontal areas in the brain, and in particular the theta frequency band, play in conflict analysis at the stimulus/response level^14,15^, at the semantic/cognitive level^16,17^ and during cross-modal prediction error processing^18^.

To our knowledge, the neural correlates associated with the amount of haptic delay have not been well studied. A few studies on visual-motor incongruency reported that theta synchronization at the midfrontal cortex reflects conflict monitoring and resolution to modality mismatch concerning self-initiated motor movement^19^ or visual stimuli involving a motor response^20,21^. Theta oscillation was reported to be more pronounced under incongruent cross-modal stimulation as compared to congruent stimulation under visuotactile matching paradigm^22^. In a similar study, incongruent stimulation induced a significantly greater theta band activity during 300–500 ms after stimulation^23^. A recent study revealed that theta power oscillation was significantly higher at the midfrontal cortex under the presence of haptic delay during passive and active tasks relying on discrete haptic feedback^24^. However, the study focused on the detection of haptic delay in a discrete haptic feedback task.

In this work, we consider the impact of the amount of haptic delay during single (as compared to dual- or multi-tasks), active task that involve continuous haptic feedback. We study EEG correlates associated with varying levels of haptic delays, including D0 (0 ms, no delay), D1 (120 ms), D2 (250 ms), and D3 (400 ms). The four haptic delays are selected through a pilot study based on a (1 up–1 down) staircase method: D1 and D2 are one standard deviation below and above the detection threshold, respectively, and D3 is three standard deviations above the detection threshold found through the pilot study. Event-related spectral perturbation (ERSP) in the theta and alpha bands are investigated in the central and midfrontal areas to determine the effect of the amount of haptic delay. Neural results are complemented with behavioral data.

## Methods

### Participants

Thirty-four subjects have been invited to participate in the experiment (17 females), where the majority of them (97%) are undergraduate students aged between 18 and 25 years. All participants were right-handed and used their right hand to complete the task. Exclusion criteria include participants below the age of 18 and/or left-handed with reported traumatic brain injuries, neural abnormalities, and/or muscle atrophy. Around 82% of the participants had no prior experience using a haptic device. The protocol followed in this study was approved by New York University Abu Dhabi Institutional Review Board (IRB: # HRPP-2021-17) and was in accordance with the Declaration of Helsinki, following its guidelines and regulations. All participants signed an informed consent form in accordance with the IRB ethics before enrolling in this study. Each participant received an Amazon voucher worth 30 dollars (USD) for their participation. Several preventive and cautionary measures were taken to protect participants from COVID-19, such as: maintaining physical distancing, adhering to surgical masks and gloves before any interaction with the participants, disinfecting the haptic device after every use and completing a symptom check form to ensure participants are symptoms free.

### Experimental setup and task

**Figure 1:**
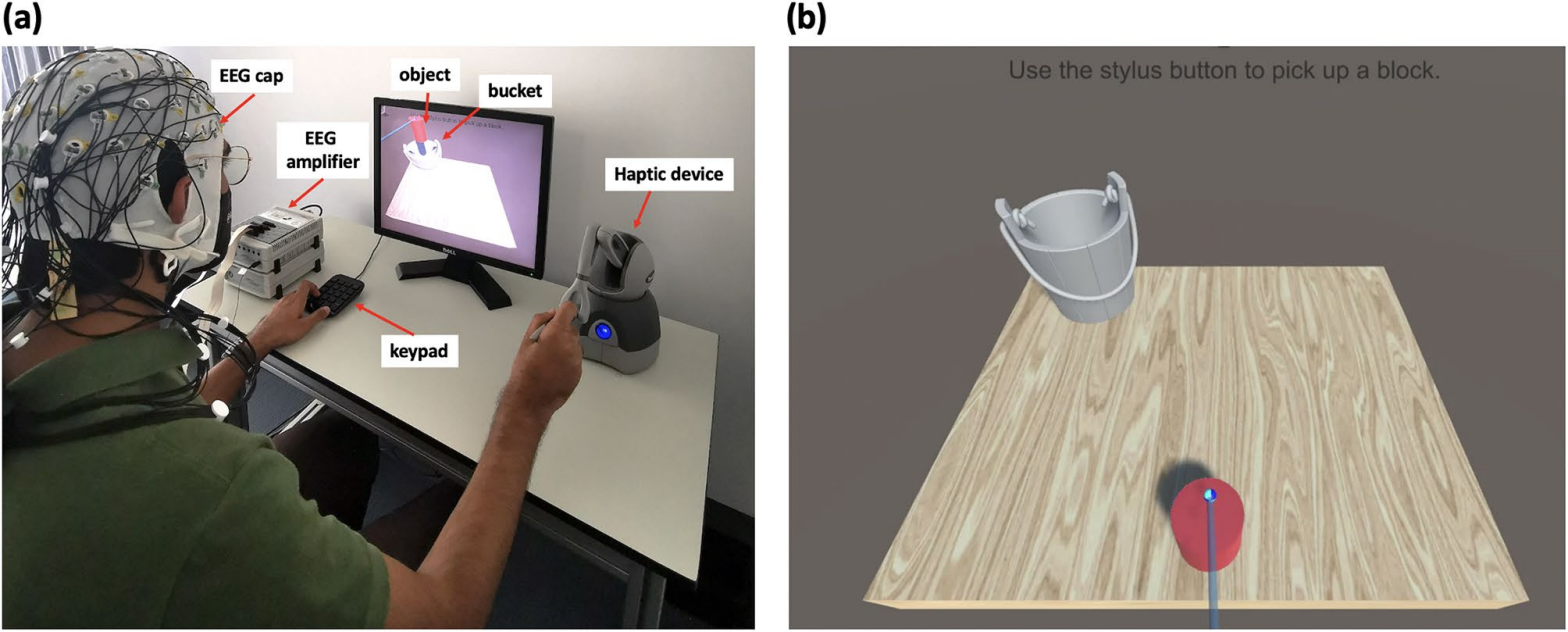
A 64-channels EEG cap is placed on the participant’s head. Wet electrodes are connected to the EEG amplifier. The participant controls the movement of the stick shown on the screen to pick up the object using the haptic device. The participant holds the stylus of the haptic device with their right hand to control the movement of the stick shown on the screen. The participant’s thumb is placed on the button integrated into the stylus, making themselves ready to pick up the object. A keypad was placed in front of the participant to answer whether they felt a delay after every trial. The participant is trained on using the haptic device with minimal body movement to avoid excessive EEG artifacts. (**b**) A screenshot of the assigned task showing the object, bucket, and stick used for picking up the object.

Participants performed a simulated pick and drop task in which the user was asked to grab a cylindrical-shaped object with their right hand and release it in a bucket using a haptic device (Geomagic Touch, 3D systems, United States). Figure 1a shows one of the participants correctly holding the stylus of the haptic device along with the experimental setup. Both haptic and visual feedbacks were provided; the visual feedback was delivered through the computer screen while the haptic feedback was delivered through the haptic device. The stylus of the haptic device is used to control the movement of a stick shown on the screen, which is in turn used to grab the object. The object is grabbed by pressing a button at the end of the stylus; once the object is grabbed, force feedback that is equal to the object’s weight is delivered. The haptic feedback is enabled as long as the button is pressed. The object should be carried towards the bucket and released by releasing the button on the stylus. Once the button is released, the object falls down visually on the screen. However, the force feedback continues to be enabled for an additional period of time, depending on the condition. We refer to this period of time as a haptic delay. Four different levels of haptic delay are provided (D0 = 0 ms, D1 = 120 ms, D2 = 250 ms, D3 = 400 ms). Those delay levels were obtained after conducting a pilot study. Figure 1b shows a screenshot of the designed task. Participants were trained on using the haptic device with minimal body movement to avoid excessive motor EEG artifacts. Once the participant was ready, ten runs were conducted. Each run consisted of 16 grab and release trials (four different levels of haptic delay $\times$ four repetitions) sequenced in a counterbalanced order by a Latin square to avoid order or sequence effects. The ten runs were separated by nine breaks with durations that are participant dependant. Those breaks were essential to avoid boredom and lack of attention during the experiment. The complete experiment took between 45 min and 1 h including the time needed to prepare the setup and place the electrodes. Overall, each participant performed 160 trials divided equally between the the four delay conditions.

**Figure 2:**
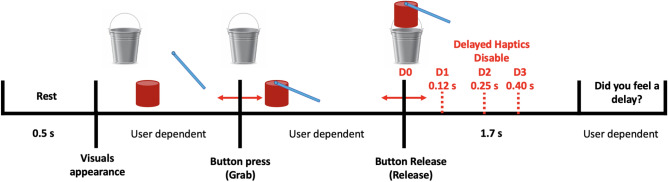
Schematic representation of the experimental task. Once the object is released, the haptic feedback represented by the object’s weight continues to be enabled under the presence of haptic delay. This delay could be either 120 ms, 250 ms, or 400 ms. Under the absence of haptic delay, the haptic feedback is disabled once the object is released, and thus, the visual and haptic modalities are in synchrony. After every trial, participants indicated whether they felt a delay or not.

Each participant had a release score which reflected the number of trials the participant successfully released the object inside the bucket. Recording this score ensures that participants perform the task with vigilance and care, as typically done in a real-life scenario. At the end of each trial, participants were prompted to rate the level of the delay they experienced in the previous trial through a keypad in a binary fashion (felt delay or did not feel delay). Participants were not informed of the actual number of delay levels they would experience during the experiment. Out of these answers, the detection ratio will be calculated to estimate the psychometric function of the haptic delay. A single trial consisted of a 0.5 s rest period during which a blank screen was presented, followed by a single grab and release move; Fig. 2 shows the sequence of events in a single trial. In addition, participants were asked to fill a post-experiment questionnaire to capture their perceptual experience. The task was developed using Unity game engine version 2018.4.5f1 (Unity technologies, United States) and Openhaptics Unity toolkit (3D Systems, United States).

### Pilot study

A pilot study was conducted to identify the haptic delay levels for the experiment. The aim was to choose delay levels that could form a proper psychometric function for the haptic delay. A psychometric function is a model used in detection and discrimination tasks to model the relationship between perceived stimulus (i.e., amount of haptic delay) and its detection rate. A properly designed experiment should lead to an S-shaped psychometric function. If the chosen haptic delay levels are far beyond the detection threshold, subjects will always perceive the haptic delay leading to a horizontal line function (constant). A proper choice of haptic delay levels should yield a psychometric function centered around the detection threshold. Nine participants took part in the pilot study. The study consisted of two sessions, each having 20 trials; each trial follows the same sequence of events shown in Fig. 2. We followed a bottom-up approach in the first session such that the first trial had zero haptic delay. In the second session, we followed an up-bottom approach such that the first trial had a haptic delay of 600 ms. We chose the staircase method (1-up 1-down) with adaptive step size^25^ to control the variations of haptic delay after each trial. The initial step size was set to 80 ms. After every inflection point, we reduced the step size by 20 ms. The haptic delay of the last five trials is recorded and averaged for each session and participant. This delay is an estimation of the 50% point on a psychometric function. In the bottom-up session, the mean of the twentieth trial delay was 150 ms with a standard deviation of 50 ms, while in the up-bottom session, the mean of the twentieth trial delay was 220 ms with a standard deviation of 80 ms. The grand average of both means is $M=$ 185 ms, and the grand average of both standard deviations is $SD=$ 65 ms. Thus, and based on this result, we chose the delay levels as below:

$$\begin{aligned}&{\hbox {D}0 : 0 \hbox {ms}\, (\hbox {probability to feel delay}< 5\%)} \\&{\hbox {D}1 : M - SD = 185 - 65 = 120 \hbox {ms}\, (\hbox {probability to feel delay}< 30\%)} \\&{\hbox {D}2 : M + SD = 185 + 65 = 250 \hbox {ms}\, (\hbox {probability to feel delay} < 70\%)} \\&{\hbox {D}3 : M + 3 \times SD =185 + (65 \times 3) = 380 \hbox {ms}\, (\hbox {probability to feel delay} > 95\%)} \end{aligned}$$

We intended to have the fourth level always perceivable (100% detection rate) and hence decided to increase the fourth level to 400 ms.

### EEG data acquisition

EEG data were recorded during the experimental session at a sampling rate of 1 kHz using BrainAmps amplifier (BrainAmps Standard, Brain Products, Germany). The Brain Vision Recorder software (BVR; Version 1.21.0201 Brain Products, Germany) was used to control the acquisition process and monitor the connection quality of the electrodes. We used a 64 Ag/AgCL based active electrodes with built-in readout circuitry, including noise cancellation and amplification. We followed the 10–20 international positioning system for placing the EEG cap on participants’ heads such that the Cz electrode lies at the vertex of the head. The ground electrode was placed at the FPz location, while the online reference was placed at FCz. To ensure high-quality signal recordings, we maintained a connection impedance between the electrode and the scalp below 15 k$\Omega$ for all the electrodes.

### EEG data pre-processing

Pre-processing and analysis of the data were performed offline using MATLAB release 2021a (MathWorks, United States) and EEGLAB toolbox (v14.1.2)^26^. A sinc FIR filter that uses a Hamming window was used to filter the data between 0.1 and 50 Hz. A notch filter was further used to suppress power line noise centered around 50 Hz. The Artifact Subspace Reconstruction (ASR)^27^ method was applied on the EEG data to remove high-amplitude artifacts such as muscular activity, eye blinks, and movements and to detect and reject highly contaminated channels. The following parameters were used for the ASR method (argflatline = 10, arghighpass = [0.025 0.075], argchannel = 0.8, argnoisy = 4, argburst = 20, argwindow = ‘off’). EEG data were then re-referenced using the Common Average Referencing (CAR) method^28^, and the data of the online reference FCz were retained.

The time between pick up and release ($\Delta$) was calculated for each trial signifying their length. Very short trials indicate that the participant picked and released the object right away, probably by mistake. A well-trained subject can take between at least 2 and 3 s to complete the pick and release task. Thus, very short trials were not acceptable, and those with $\Delta$ $\le$ 1 second were rejected. The rest of the trials were then epoched from − 2 to 2.7 s around the actual release of the object event. Independent component analysis (ICA) was further applied to isolate and remove any remaining ocular and muscular artifacts.

### Electrodes, bands and time-windows selection

We based our selection of electrodes, time windows, and frequency bands on the currently available literature Based on our previous study^24^, we found that there is a statistically significant difference in the theta band at the midfrontal cortex between synchronous stimulation (visual and haptic) and delayed haptic stimulation^24^. In addition, supporting literature is found on the role that midfrontal and central theta plays in multisensory attention and conflict processing, and resolution^29^. Most of the literature work focuses on analyzing data from the FCz electrode, and its surrounding^20,30^. Therefore, in this work, we examine theta band at the midfrontal region of interest (ROI) comprising the following electrodes: FC1, FCz, FC2, F1, Fz, F2. We also found some evidence in the literature on the role alpha de-synchronizat ion plays in sensory information processing and cross-modal matching in the parietal region^31,32^. We consider the following electrodes in the parietal ROI to examine alpha de-synchronization: P1, Pz, P2. In addition to the cognitive correlates, we examine sensory correlates at the cortical area that processes sensorimotor stimuli, namely the contralateral sensorimotor ROI. The impact of the haptic delay is examined, particularly in the beta band. Beta band power is normally modulated during movement, and post-movement of the limbs^33^. We focus our analysis on FC1, FC3, C1, C3, CP1, CP3 electrodes as they lie over the sensorimotor cortex, and they are known to best capture the movement-related neural activation^34,35^. All topography plots shown in the manuscript are averaged over time-windows identified where the neural activation is found to be maximal.

### EEG data analysis

Epoched data was spatially filtered using the surface Laplacian transform (m-constant: 4, head radius: 10 cm, smoothing constant: 10$^{-5}$) using functions in the CSD toolbox in MATLAB (version 1.1)^36^. It has been shown that applying CSD transform on epoched data improves the resolution of spatio-temporal features of the EEG data^37,38^. For the ERSP realization, the filtered epoched data was transformed to the time–frequency domain using Morlet wavelet transformation with a cycle range that increases logarithmically from 4 to 10 to maintain a high resolution at higher frequencies. We used 50 frequencies logarithmically spaced between 1 and 50 Hz; the logarithmic scale was used to highlight the low-frequency activation which is the focus of this study. The power of the transformed signals is then calculated by squaring their amplitude. Epochs of the same condition were then averaged. In other words, the mean was used as a central tendency measure to represent the ERSP data of each participant. Decibel conversion was used to normalize the power time series. The dB correction was done on the trial-average basis such that the condition-average baseline is used. Finally, the power of theta (3–8 Hz), alpha (8–13 Hz), and beta (13–30 Hz) was extracted, which are the bands of our interest. We consider the time window between 0 and 1000 ms for the theta, alpha, and beta-band oscillations. The choice of the baseline under the complex setting of the experimental paradigm was not very straightforward. The rest period is not precisely before the release event and the time between the rest period and the release event is user dependent. The time period before the release event is not exactly neutral, however, the task timeline right before the release event is identical across conditions; thus we could use it as a baseline to highlight the effect of the haptic delay only. Other studies in the literature which had complex experimental protocols used baselines right before the onset despite the fact they were not completely neutral^24,39,40^. We chose the time period that is − 900 to − 200 ms before the button release event as a baseline period for dB normalization for the following reasons: (1) away from the onset to avoid the temporal smoothing inherent in wavelet transform^41^; (2) long enough to accommodate a few cycles of the lowest frequency under consideration (i.e.: 3 Hz)^42^; (3) far from the edges of the epoch (epoch length is − 2 to 2.7 s around the onset)^42^. (4) Contained between the minimum grab and release duration (1000 ms).

### Statistical analyses

Randomization tests are ideal for EEG data statistical analysis since they make no assumptions on the data distribution. This feature is particularly useful in the biomedical field, where group sizes are typically small. Another advantage is that randomization tests are more generic as they do not rely on a particular test statistic on which the statistical inference is based upon (i.e., F-statistic, t-statistic or z-score)^43^. For these reasons, we used nonparametric randomization-based ANOVA method to compare group differences. All *p* values associated with EEG data in this manuscript described as statistically significant are less than or equal to 0.05 (2-tailed). For ERSPs, statistical analysis was carried out by first splitting the time window of interest to 50 equally sized intervals (each is 20 ms). The neural correlates of interest were averaged per interval, and the statistical inference was conducted on each of the time bins to identify any statistically significant difference between the conditions. We first identify the time intervals during which statistically significant neural activation is found across the four conditions. If any of the conditions differ significantly from the overall group mean, then the test shall report a significant statistical difference for that interval. Then, we compare each pair of conditions. In total, we conducted 12 tests to compare every pair of conditions for the theta, alpha, and beta bands oscillations. The analysis is conducted on the group level such that trials from the same participants are averaged and are considered as a single point in the sample. Sensory and cognitive correlates were split into 20 ms time windows, and oscillations were averaged out within every time bin. To correct for multiple comparisons and to limit type 1 error, we corrected all reported *p* values using the false discovery rate (FDR) method^44^. The number of comparisons equals the number of time bins used during the statistical analysis, which is 50 for the ERSP analysis. Throughout the manuscript, we report the corrected *p* values as well as the time range during which the statistically significant difference was found.

Statistical analysis on the behavioral data involved using randomization-based ANOVA test to verify if there is any statistically significant difference found in the proportion of detection between pairs of all conditions. All *p* values in this manuscript associated with behavioral data described as statistically significant are less than or equal to 0.005 (2-tailed).

## Results

### Behavioral data and questionnaire

**Figure 3:**
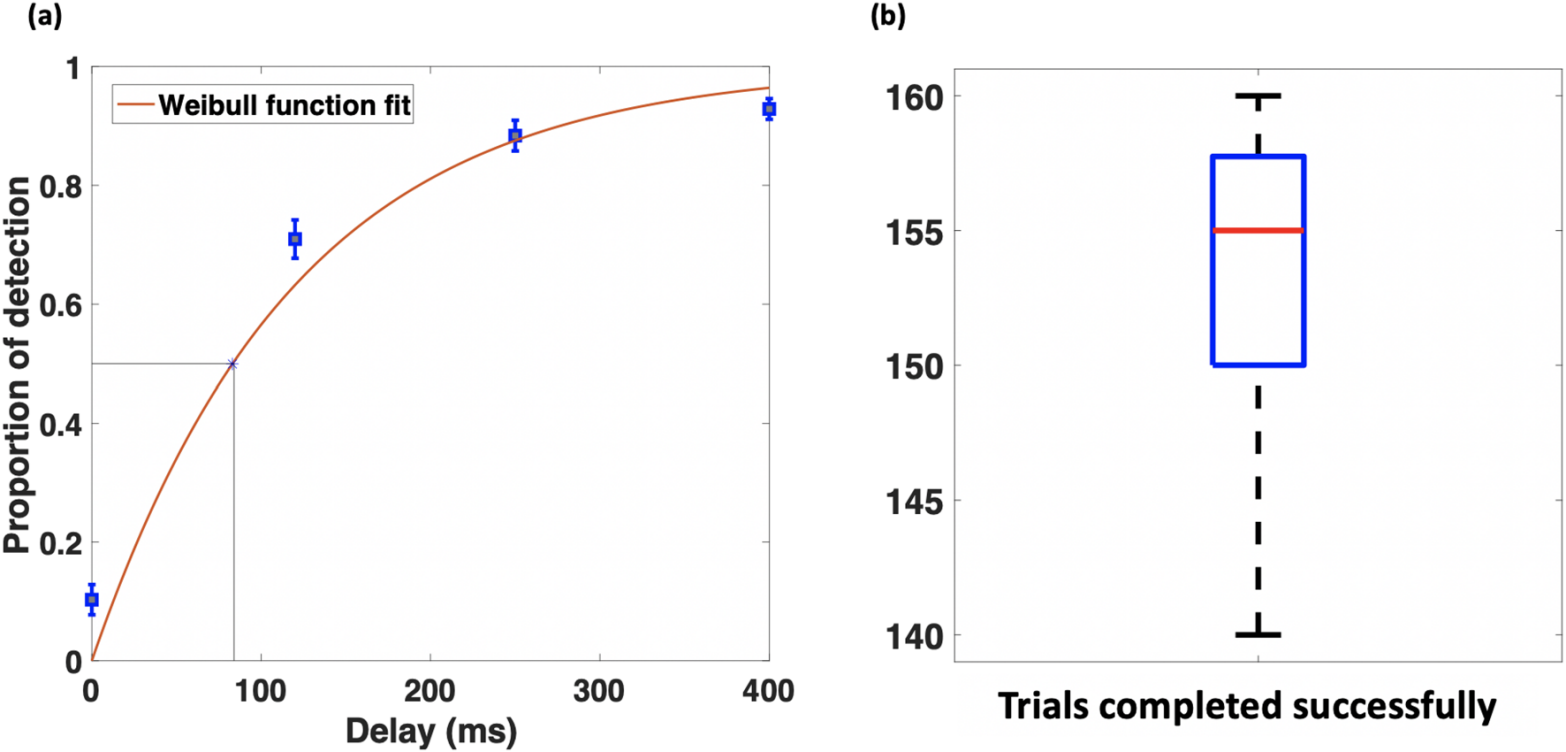
(**a**) The proportion of detected trials per condition are plotted and fit to a Weibull function forming a psychometric function for the haptic delay. The 50% detection threshold is estimated and marked to be around 85 ms. Error bars represent the standard error of the mean. (**b**) Boxplot showing the trials completed successfully by participants. A successful trial is identified by placing the object inside the bucket.

Participants were asked after each trial whether they could perceive a haptic delay. The detection proportions of all subjects and trials were calculated per condition and can be seen plotted in Fig. 3a. To estimate the psychometric function of the haptic delay, we used Weibull cumulative distribution function commonly used for this purpose^45,46^. We could estimate the 50% detection threshold of the haptic delay from the estimated psychometric function. The 50% detection threshold is defined as the shortest delay that can be detected half the time on the psychometric function. At a 0.5 detection rate, the delay is around 85 ms, which is the estimated threshold. The error bars represent the standard error of the mean. Participants performed the task with vigilance as can be seen from Fig. 3b. All participants completed at least 140 trials out of 160 successfully in a sense they released the object inside the bucket.

Additionally, participants had to answer a post-experiment questionnaire to capture their subjective experience. As part of the questionnaire’s questions, participants attempted to guess the number of delay levels they had experienced during the experiment (no choices are provided). Results showed that 18 % reported they experienced three levels, 44 % reported they experienced four levels, 32 % reported they experienced five levels, and 6 % reported they experienced more than five levels. Overall, 95 % of participants reported they could distinguish between the different haptic delay levels and they were attentive to whether a haptic delay was perceived. We used randomization-based ANOVA test to compare the proportion of detection for the different pairs of delay conditions highlighted in Fig.3a. There was found a statistically significant difference in the proportion of detection between all the pairs of conditions [$\textit{p}$ $\le$ 0.005, randomization-based ANOVA test].

### Midfrontal theta oscillation

**Figure 4:**
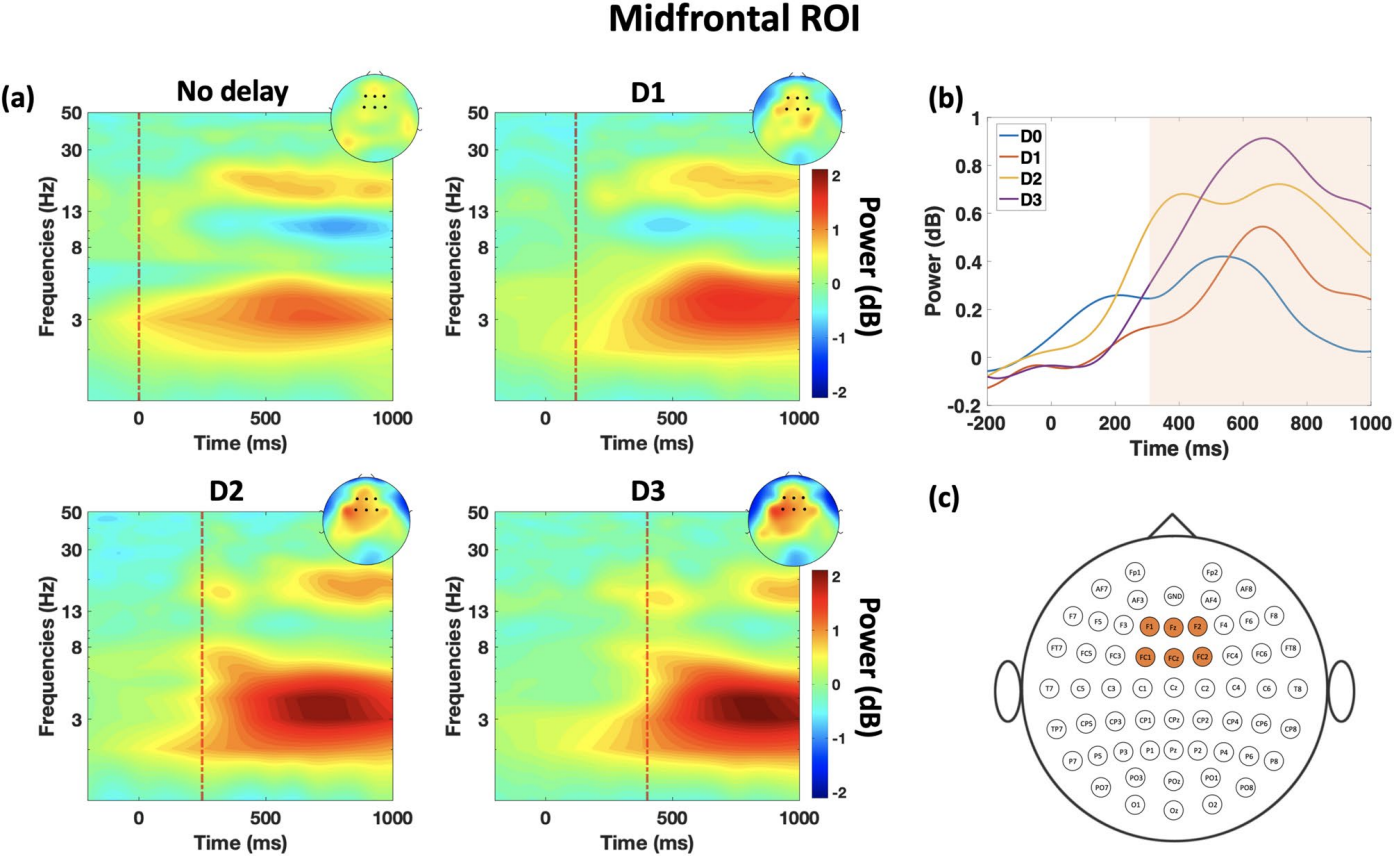
Time–frequency representation of theta band correlates at the midfrontal ROI (**a**) ERSP dynamics of the EEG data in the midfrontal ROI averaged over trials and subjects per condition. The dashed red line shows the event of the haptic feedback disable. Topography plots are obtained by averaging power in the window of 400–900 ms where the maximal effect of midfrontal theta is observed. (**b**) Time-course of the theta power under the four delay conditions. Highlighted region signifies the time interval during which a statistically significant difference is found between the four conditions ($\textit{p}$ $\le$ 0.05, randomization-based ANOVA test, FDR corrected; $t_{range}$ = [300–1000 ms]). (**c**) Channels included in the theta band analysis at the midfrontal ROI.

A modulation in the midfrontal theta power is observed when a haptic delay is introduced. Figure 4a shows the ERSP dynamics of the theta band for the four conditions under consideration at the midfrontal ROI. An examination of the time–frequency EEG data at the midfrontal ROI, highlighted in Fig. 4c, revealed theta band synchronization in all four conditions with varying intensity and frequency ranges. Both intensity and frequency range increase with the increase of haptic delay. When no delay is introduced (synchronous condition), theta synchronization is barely detected at the low theta range ($\le$ 5 Hz). On the contrary, theta synchronization was clearly observed with longer delays for D3 (3–8 Hz) and for D1 and D2 as well ($\le$6 Hz). Temporally, theta synchronization peaks between 400 and 1000 ms for all the four conditions. The peaking behavior of theta synchronization, shown in Fig. 4b differs from one condition to the other. The duration at which there is statistically significant difference between the conditions is highlighted in Fig. 4b ($\textit{p}$ $\le$ 0.05, randomization-based ANOVA test, FDR corrected; $t_{range}$ = [300–1000 ms]). Table 1 lists the time-intervals where a statistically significant difference was found ($\textit{p}<$ 0.05) between each pair of conditions.

**Table 1:** Time intervals where a statistically significant difference was found between the time-course of theta power of the different delay conditions [*p* < 0.05, randomization-based ANOVA test, FDR corrected].

Condition	D1	D2 (ms)	D3
D0	0–220 ms and 660–1000 ms	280–1000	0–260 ms and 480–1000 ms
D1	–	160–1000	420–1000 ms
D2	–	–	0–420 ms

### Parietal alpha oscillation

**Figure 5:**
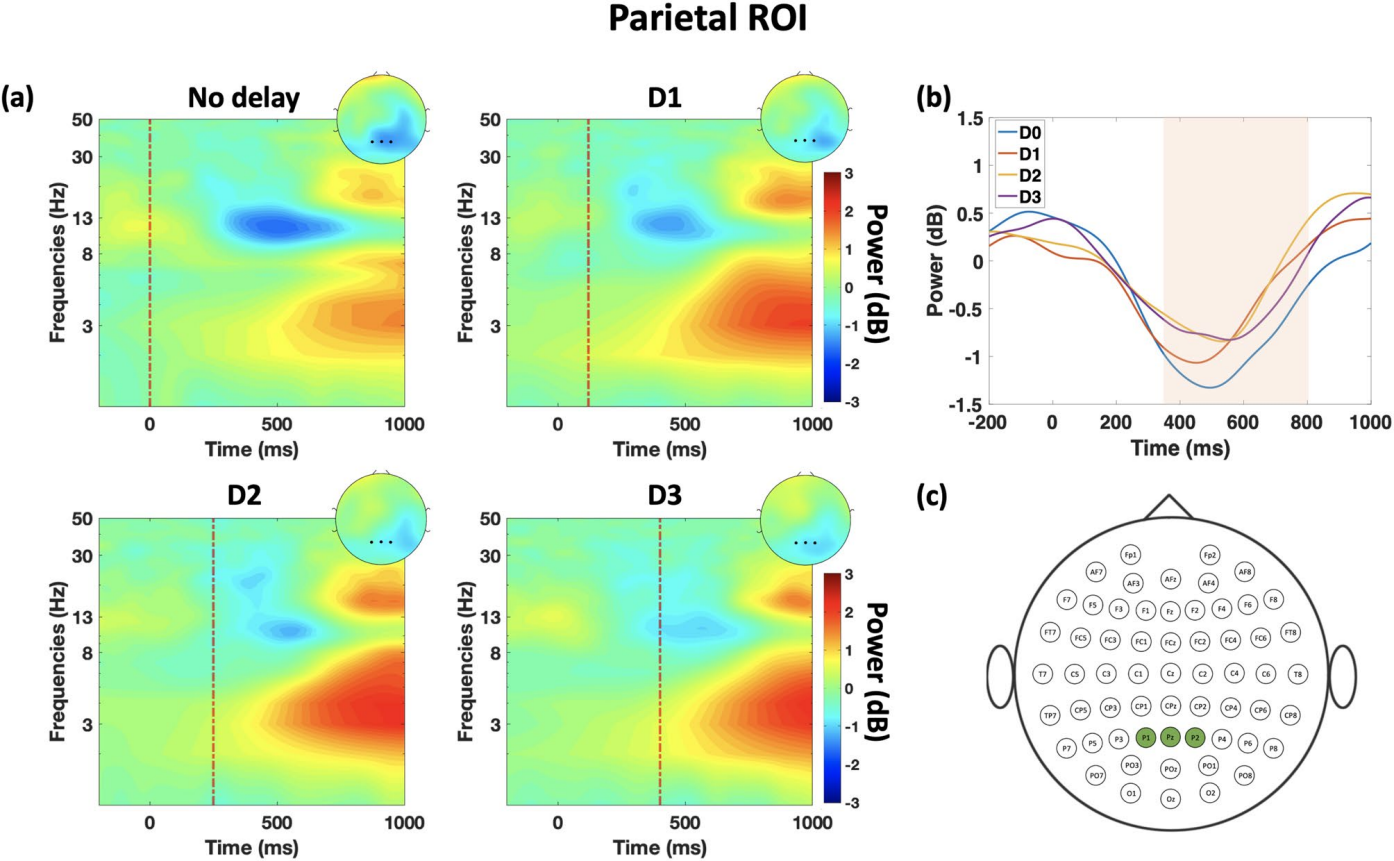
Time–frequency representation of alpha band correlates at the parietal ROI (**a**) ERSP dynamics of the EEG data in the parietal ROI averaged over trials and subjects per condition. The dashed red line shows the event of the haptic feedback disable. Topography plots are obtained by averaging power in the window of 300–700 ms where the maximal effect of alpha desynchronization is observed. (**b**) Time-course of the alpha power under the four delay conditions. Highlighted region signifies the time interval during which a statistically significant difference is found between the four conditions ($\textit{p}$ $\le$ 0.05, randomization-based ANOVA test, FDR corrected; $t_{range}$ = [340–800 ms]). (**c**) Channels included in the alpha band analysis at the parietal ROI.

Alpha power at the parietal ROI is observed to be modulated under the presence of haptic delay. Figure 5a shows the ERSP dynamics of alpha power for the four conditions. Descriptively, alpha desynchronization is stronger in the synchronous condition, that is in the absence of delay, compared to the cases where haptic delay is present. On the contrary, there seems to be no difference in alpha desynchronization among the different delay conditions. The time course of alpha power suggests that desynchronization peaks between 300 and 700 ms regardless of the condition. Statistically speaking, the duration at which there is a statistically significant difference between the conditions is highlighted in Fig. 5b ($\textit{p}$ $\le$ 0.05, randomization-based ANOVA test, FDR corrected; $t_{range}$ = [340–800 ms]). From Table 2, we infer that there is no significant difference in alpha desynchronization between the different delay conditions. At the same time, there is a significant difference between each of the delay levels and the synchronous condition. In other words, there is a binary effect on the alpha power such that when a haptic delay is introduced, a decrease in alpha desynchronization is observed. The electrodes used in this analysis as part of the parietal ROI are highlighted in Fig. 5c.

**Table 2:** Time intervals where a statistically significant difference was found between the time-course of alpha power of the different delay conditions [*p* < 0.05, randomization-based ANOVA test, FDR corrected].

Condition	D1 (ms)	D2	D3
D0	540–760	320–1000 ms	420–580 ms
D1	–	*p* > 0.05	*p* > 0.05
D2	–	–	*p* > 0.05

### Sensorimotor beta oscillation

**Figure 6:**
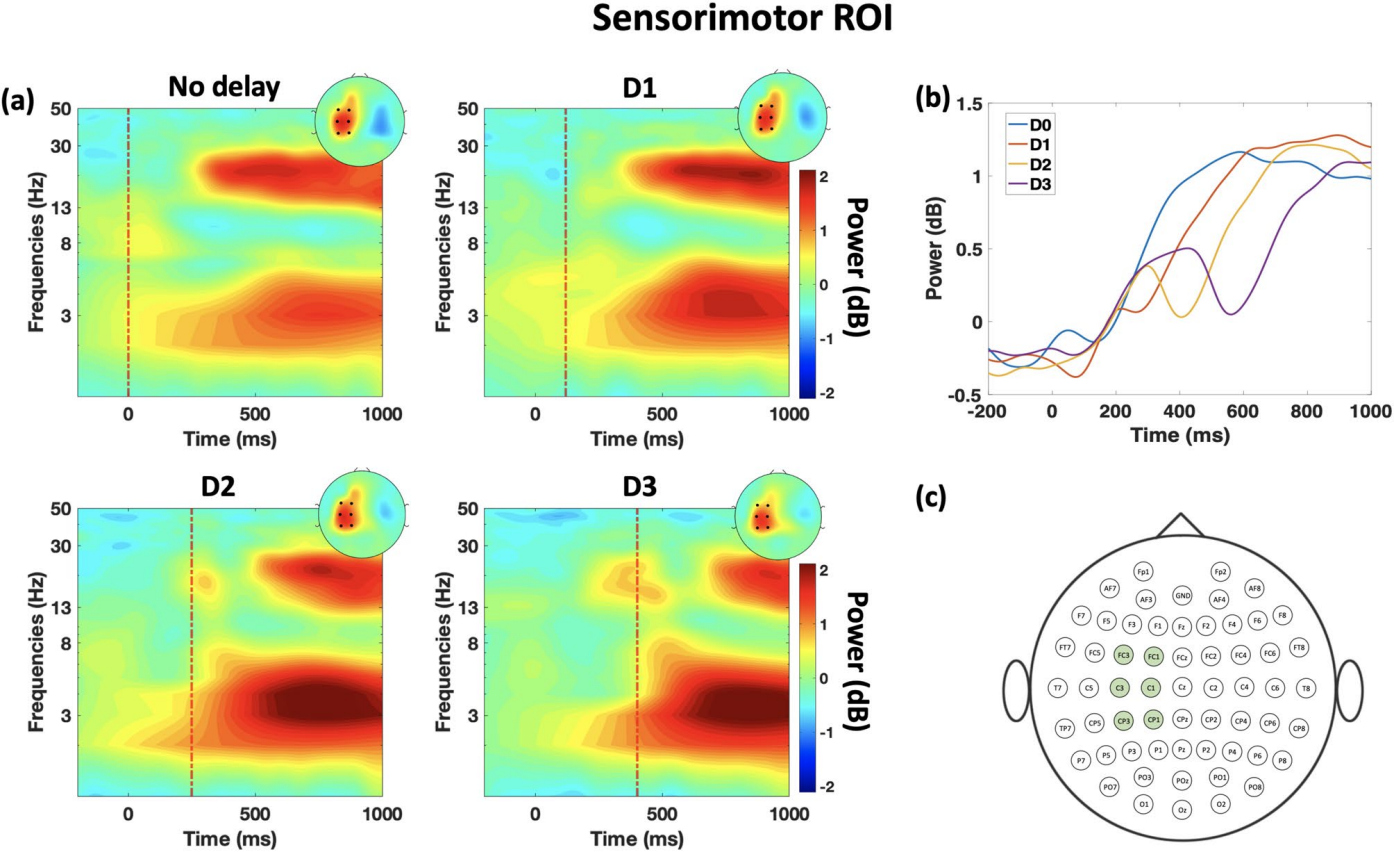
Time–frequency representation of the sensory correlates at the sensorimotor ROI. (**a**) ERSP dynamics of the EEG data in the sensorimotor ROI averaged over trials and subjects per condition. The dashed red line shows the event of the haptic feedback disable. Topography plots are obtained by averaging power in the window of 400+*delay*–600+*delay* ms where the maximal effect of beta rebound is observed. (**b**) Time-course of the beta power under the four delay conditions. The compounded beta rebound can be observed particularly clear at D2 and D3. (**c**) Channels included in the sensory correlates analysis at the sensorimotor ROI.

Post the release of the object, beta synchronization is observed under all the delay conditions. Figure 6a shows the time–frequency representation of beta power at the sensorimotor ROI which is highlighted in Fig. 6c. As can be observed from Fig. 6a, beta synchronization is clear at the contralateral sensorimotor region. It can be seen from Fig. 6a,b that there is an increase in the beta power that occurs twice during the time course of the trial; we call this effect a compounded beta synchronization, particularly clear at D2 and D3 conditions. Compounded beta synchronization consists of two consecutive synchronizations and thus the name. The first increase in beta power occurs shortly after the onset (thumb release) while the second increase is tied to the haptic release event. It is observed from Fig. 6a that the second increase in beta oscillation is time-locked to the disable of the haptic feedback (dashed line) and tied to the amount of haptic delay. Apart from the delay observed in the second beta synchonization, no other type of modulation is observed in the beta power or the rate of power increase.

## Discussion

This work investigated the neural correlates associated with the amount of haptic delay during continuous haptic feedback as part of a simulated pick and place task. The main findings were that parietal alpha showed a significant modulation that encodes the presence of haptic delay whereas midfrontal theta oscillation plays a pivotal role in quantifying the amount of haptic delay. In the following, we discuss the implications of each of these modulations and their role in encoding the presence and amount of the haptic delay.

Based on the behavioral data in Fig. 3a, some participants falsely perceived a delay when there was actually not (synchronous condition). Similarly, at a delay of 400 ms, which is way beyond the 50% detection threshold, some participants occasionally reported not feeling a delay. Both of these cases show that participants, rarely, had perceptual illusions. In this regard, perceptual illusions have been reported to occur due to prior expectations^47^ or lack of attention^48^. Given the nature of EEG-based studies and the task repetition involved, it is inevitable that participants attention could be diverted in a few trials causing illusory perception. We counteracted this effect by questioning the participant at the end of every trial whether they perceived a haptic delay. This is to ensure that participants are paying attention to the parameter under study (delay) and they are not skipping trials. We also offered a sufficient number of breaks during the experiment to minimize fatigue and boredom^49^. Statistically speaking, it was found that there is a significant difference between the proportion of detection across all the pairs of delay conditions. This shows that participants tended to identify perceptual differences between the conditions from a behavioral perspective.

From a neural perspective on the other hand, statistically significant differences were also found in the theta band power across all the pairs of conditions. In a previous study^24^, we found that midfrontal theta power encodes the presence of haptic delay under discrete haptic feedback settings. In particular, the study found spectral leakage in the theta effect under delayed-discrete haptic feedback condition during an active movement task, where the introduced delay was 220 ms. In the current study, the activation is contained within the theta band under the synchronous and delayed conditions regardless of the amount of the delay. One possible explanation is the difference in the nature of the haptic delay. In this study, the introduced delay was in the disabling of the continuous haptic feedback, whereas in the cited study, the introduced delay was in the enabling of a discrete haptic feedback. In this light, it is tempting to state that the nature of the haptic feedback (discrete vs. continuous) and the nature of the delay (enable vs. disable) could play a role in the spectral component of the midfrontal theta activation.

In line with previous findings^50^, we found a neural feature that is associated with the detection of haptic delay during continuous haptic interaction. A significant difference in the alpha desynchronization was only observed between the pairs of synchronous condition (D0) and every other delay condition (D1–D3). A very interesting implication of this result is the possible relation between the increased alpha power and visuomotor prediction error (PE). In a visumotor task that involves visual errors with respect to a motor movement, Savoie et al.^32^ showed that an increase in parietal alpha power is associated with visuomotor PE. Despite some similarity between the two studies, a notable difference is that the current study tackles the mismatch between the predicted and actual motor consequences of the visual stimulus. This could indicate that whether the mismatch is detected in the visual modality or the haptic/motor modality, the neural manifestation in the parietal alpha seems similar.

In the sensorimotor contralateral ROI, beta activation was temporally modulated with the different levels of haptic delay (see Fig. 6a,b). Event-related desynchronisation (ERD) is observed before active and during active and passive movements of limbs^51^ while event-related synchronisation (ERS) is observed upon the termination of the movement regardless of its type^33^; this particular type of synchronization is commonly referred to as post-movement beta rebound (PMBR). PMBR is generally identified by a pronounced increase in beta power within 1 second upon movement termination^33^. Analyzing the participant’s hand movement during a single trial of the task, there are two movements involved; the first is the active movement performed upon releasing the object (moving the thumb up from the stylus) which occurs at t = 0. Upon the termination of the thumb movement, a clear ERS is observed around the same time under all conditions. The second movement is a passive one in which an upward force feedback is applied on the participant’s hand when the object is released. This second force occurs at either t = 0 ms, t = 120 ms, t = 250 ms, or t = 400 ms depending on the delay condition. The ERD is observed during the occurrence of this force feedback, followed by a PMBR upon the passive movement termination. This explains the valley feature observed in Fig. 6b which is delayed proportionally with the introduced delay. Considering the time-period before the haptic release as a baseline, we could assume that the increase in beta power right after is indeed a beta rebound phenomenon^33,52,53^. Interestingly, the increase in beta oscillations (second rebound) caused by haptic feedback appeared to be greater than the increase in beta oscillations (first beta rebound) caused by the cessation of the actual motor task (dragging and dropping an object). More research is needed to determine whether beta rebound is a phenomenon that occurs simply after a physical motor task is performed or a phenomenon that occurs when the completion of motor performance is cognitively detected.

One limitation of this study is that all the delay conditions were set to predefined constant values. In other terms, variance of delay or jitter effect is not considered in the current study. Given the significant impact of jitter on the quality of user experience^54^, future work should examine neural correlates associated with jitter. Furthermore, given that all participants were recruited from one age group (18–25 years old), the results are not generalizable to other age groups (given how haptic perception varies across age groups^55^).

## Data Availability

The EEG dataset analysed and discussed in this work can be obtained from the following link on OSF: https://osf.io/fdzns/.
